# Neurocircuitry of Deep Brain Stimulation for Obsessive-Compulsive Disorder as Revealed by Tractography: A Systematic Review

**DOI:** 10.3389/fpsyt.2021.680484

**Published:** 2021-07-01

**Authors:** Eduardo Varjão Vieira, Paula Ricci Arantes, Clement Hamani, Ricardo Iglesio, Kleber Paiva Duarte, Manoel Jacobsen Teixeira, Euripedes C. Miguel, Antonio Carlos Lopes, Fabio Godinho

**Affiliations:** ^1^Division of Neurosurgery, Department of Neurology, University of São Paulo Medical School, São Paulo, Brazil; ^2^Department of Radiology, University of São Paulo Medical School, São Paulo, Brazil; ^3^Division of Neurosurgery, Sunnybrook Health Sciences Centre, Harquail Centre for Neuromodulation, Sunnybrook Research Institute, University of Toronto, Toronto, ON, Canada; ^4^Department of Psychiatry, University of São Paulo Medical School, São Paulo, Brazil; ^5^Functional Neurosurgery, Santa Marcelina Hospital, São Paulo, Brazil; ^6^Center of Engineering, Modeling, and Applied Social Sciences, Federal University of ABC, Santo André, Brazil

**Keywords:** deep brain stimulation, tractography, obssesive compulsive disorders, diffusion tensor imaging, white matter

## Abstract

**Objective:** Deep brain stimulation (DBS) was proposed in 1999 to treat refractory obsessive-compulsive disorder (OCD). Despite the accumulated experience over more than two decades, 30–40% of patients fail to respond to this procedure. One potential reason to explain why some patients do not improve in the postoperative period is that DBS might not have engaged structural therapeutic networks that are crucial to a favorable outcome in non-responders. This article reviews magnetic resonance imaging diffusion studies (DTI-MRI), analyzing neural networks likely modulated by DBS in OCD patients and their corresponding clinical outcome.

**Methods:** We used a systematic review process to search for studies published from 2005 to 2020 in six electronic databases. Search terms included obsessive-compulsive disorder, deep brain stimulation, diffusion-weighted imaging, diffusion tensor imaging, diffusion tractography, tractography, connectome, diffusion analyses, and white matter. No restriction was made concerning the surgical target, DTI-MRI technique and the method of data processing.

**Results:** Eight studies published in the last 15 years were fully assessed. Most of them used 3 Tesla DTI-MRI, and different methods of data acquisition and processing. There was no consensus on potential structures and networks underlying DBS effects. Most studies stimulated the ventral anterior limb of the internal capsule (ALIC)/nucleus accumbens. However, the contribution of different white matter pathways that run through the ALIC for the effects of DBS remains elusive. Moreover, the improvement of cognitive and affective symptoms in OCD patients probably relies on electric modulation of distinct networks.

**Conclusion:** Though, tractography is a valuable tool to understand neural circuits, the effects of modulating different fiber tracts in OCD are still unclear. Future advances on image acquisition and data processing and a larger number of studies are still required for the understanding of the role of tractography-based targeting and to clarify the importance of different tracts for the mechanisms of DBS.

## Introduction

Obsessive-compulsive disorder (OCD) is a mental illness characterized by abnormal obsessions (repetitive, intrusive, and unwanted thoughts or images), leading to distressing and repetitive behaviors (compulsions). It affects around 2% of the population and may significantly impair quality of life (1). OCD has been considered a disorder of altered functional neural circuits involving subcortical and prefrontal cortical areas (2–4). According to a recent meta-analysis, voxel-based studies showed that OCD patients had smaller gray matter volume in the medial orbitofrontal cortex (OFC), nucleus accumbens (NAC), dorsomedial prefrontal cortex (PFC) and dorsolateral PFC (5). Functional neuroimaging studies have shown that multiple circuits are involved in mechanisms of different OCD symptoms: (i) a fronto-limbic circuit involving the amygdala and the ventromedial PFC is associated to affective responses (e.g., fear and anxiety); (ii) a sensorimotor circuit that includes the supplementary motor area, the putamen and the thalamus is related to motor behavior and sensory integration; (iii) a ventral cognitive circuit includes the inferior frontal gyrus (IFG), the ventrolateral PFC, the ventral caudate and thalamus associates with self-regulatory control of behavioral acts; (iv) a ventral affective circuit including the OFC, the NAC, and the thalamus is associated with reward processing; and (v) a dorsal cognitive circuit formed by the dorsolateral and dorsomedial PFC, the dorsal caudate and the thalamus is related to executive functions and the top-down regulation of emotions (4). It is important to note that other fronto-parietal and cerebellar circuits may also play a role (6).

Deep brain stimulation (DBS) was proposed as a therapeutic option for OCD patients refractory to conventional treatments, based on previous results of capsular lesions (7, 8). This therapy involves the delivery of electrical stimulation through electrodes implanted in the brain parenchyma. Although, far from being completely understood, multiple and not exclusive mechanisms are likely involved in the effects of DBS. These include local neuronal effects (such as somatic inhibition, axonal-dendritic activation, and neurochemical synaptic changes), in addition to wide-network effects, characterized by the disruption of electrical pathological oscillations and synaptic plasticity (9–11). After the first published trial in 1999 (12) numerous studies have been conducted suggesting that approximately 30–50% of patients fail to respond to this therapy (13–15). Potential explanations to these findings are the multiple circuits involved in mechanisms of OCD and the fact that the modulation of different cortical regions and white matter pathways (WMP) may have a distinct impact on dysfunctional circuits, leading to discrepancies in outcomes (16). WMPs, particularly those within the ventral region of the anterior limb of the internal capsule (ALIC), are organized in different functional sectors and show intrinsic anatomical variability (17, 18). Therefore, similar to movement disorders where improper placement of DBS leads may result in a suboptimal response (19), slight variations in the anatomical location of DBS contacts within the NAC/ALIC may engage distinct WMPs, yielding different outcomes in OCD. To address this issue, one study compared the anatomical position of electrode contacts between OCD patients who did or did not respond to DBS. Using a common standard space for analyses, they found no difference between these two groups (20). This raised the hypothesis that “connectomic” rather than anatomical differences across patients might be associated with therapeutic responses (20–23). Along this line, diffusion tensor imaging (DTI-MRI), a magnetic resonance imaging technique that measures the restricted diffusion of water within the tissue in order to produce images of neural tracts, may contribute with current surgical techniques to refine neurosurgical targeting.

This systematic review aims to identify potential fiber tracts and connectomic data, along with limitations and research perspectives on DBS for OCD.

## Methods

### Criteria for Considering Studies in This Review

#### Search Methods and Questions

Six databases were searched in the review: Medline, Pubmed, Google Academic, LILACS (Latin American and Caribbean Health Science Information database), EMBASE, and Cochrane Library. The following search terms were considered: “obsessive-compulsive disorder,” “deep brain stimulation,” “diffusion tensor imaging,” “diffusion-weighted imaging,” “diffusion tractography,” “tractography,” “connectome,” “diffusion analyses,” and “white matter.” These terms were associated using boolean operators. References were cross-checked to identify additional studies. Search strategy for identification of reports in Pubmed are depicted in Appendix. The primary question of this review was as follows: “which WMPs and corresponding cortical areas are modulated by DBS and associated with an optimal outcome in clinically-refractory OCD patients?”

#### Types of Studies

We included articles using MRI diffusion analyses (DTI-MRI) to identify tract targets. We started our search in 2005, when the first report of white matter changes in OCD patients was reported using DTI-MRI (24), and extended until October 2020, including articles that met the eligibility criteria presented below. Replicate studies, posters, preclinical reports, studies on surgical ablative procedures and general reviews were excluded.

#### Participants

Participants were adults of both sexes, 18 years or older, diagnosed with refractory OCD according to standard criteria (25).

#### Type of Interventions

Selected studies included tractography analysis performed on either individual OCD patients (patient-specific) or normative control data - such as the Human Connectome Project (HCP). No restriction was made regarding the following variables: (i) surgical target: NAC, ALIC, ventral capsule – ventral striatum (VC/VS), subthalamic nucleus (STN), medial forebrain bundle (MFB), bed nucleus of the stria terminalis, inferior thalamic peduncle; (ii) method of imaging acquisition and analysis (strength of the main static magnetic field, diffusion b-value, number of diffusion gradient directions, fiber tracking method, and post-processing software). Only studies that described the electrical modulation of WMPs and/or cortical areas by DBS or reported the distance between active DBS contacts and WMPs were retrieved.

#### Variables of Interest and Outcome Measures

The primary variable of interest was the WMP and cortical areas modulated by DBS. Postoperative clinical outcome was assessed with the Yale-Brown Obsessive- Compulsive Scale (Y-BOCS).

#### Data Collection and Analysis

**Figure 1:**
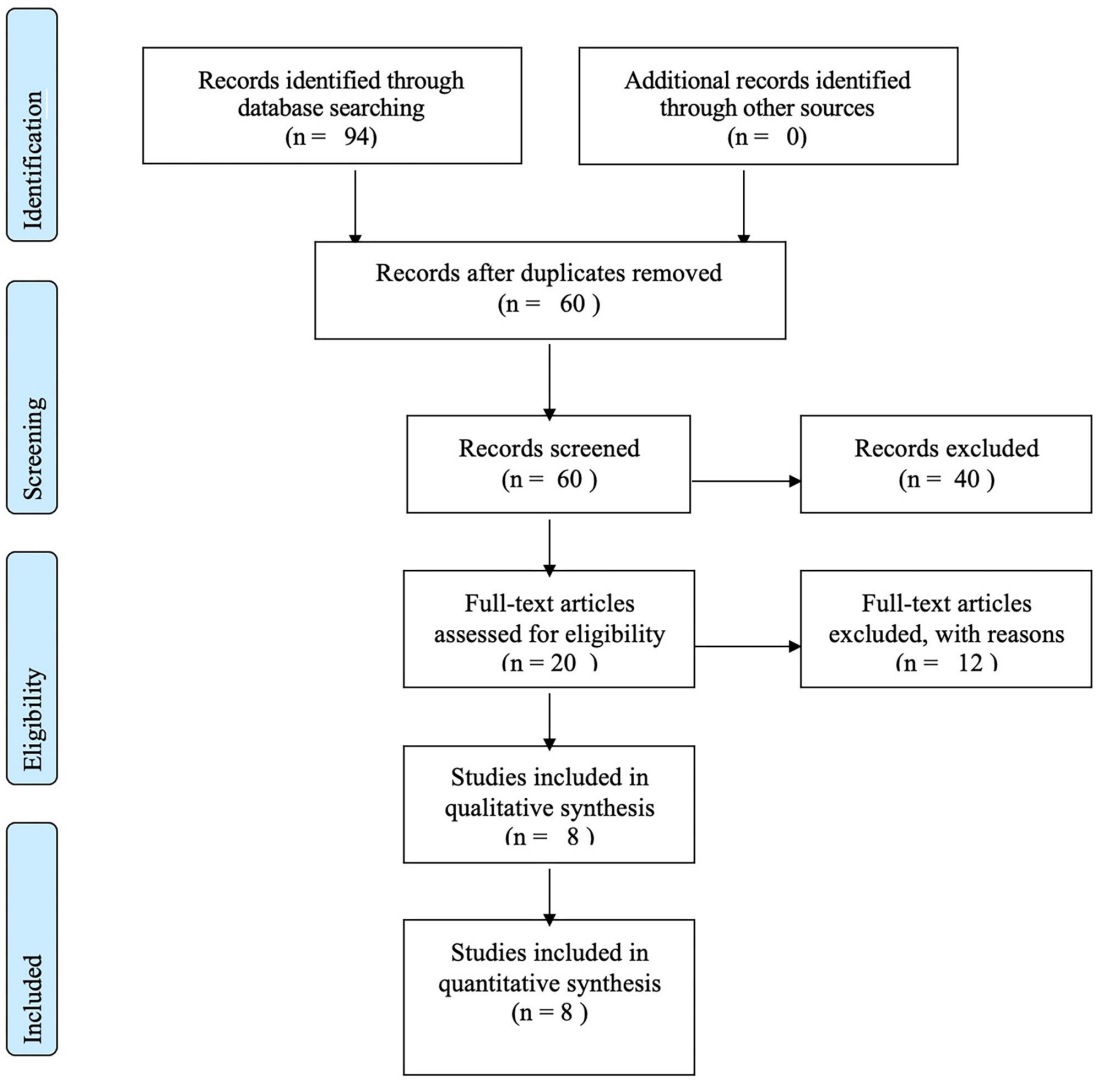
Prisma flow diagram.

The identification and selection of studies was made in two stages. First, we downloaded the studies retrieved via electronic search to a reference management database (Mendeley) and a software designed for systematic review (StArt - State of the Art through Systematic Review) (26). Duplicates were removed. Two authors (EVV and FG) independently screened the titles and abstracts to assess whether they met the inclusion criteria. Full texts of the selected references were obtained and examined. We were not blinded to the authors names, affiliated institutions, journal of publication, or trial results. Two authors (EVV and FG) independently reviewed each article for eligibility. Any disagreement was resolved by a third author (PRA). The study selection process is presented in a PRISMA diagram (Figure 1).

#### Data Extraction and Management

Two authors (EVV, FG) independently extracted study data on a pre-defined form, which was cross-checked for accuracy. Disagreements were resolved by a third author (PRA). The following data were extracted from each study: year of publication, number of patients with treatment-refractory OCD undergoing surgery, DBS target, follow-up (months), pre and postoperative Y-BOCS, neuroimaging technique (strength of the main static magnetic field, diffusion b-value, number of diffusion gradient directions, fiber tracking method, post-processing software), the WMP, and cortical regions involved in DBS effects.

#### Risk of Bias in Individual Studies

By the time of this systematic review, there were many available methods of diffusion analyses and no consensus about the best practice or reliability grading. Not all parameters were described in each study. Some of these are known to be sacrificed when increasing diffusion strength such as b-value (27).

## Results

### Description of Studies

From the 94 studies initially retrieved, we had a total of 60 abstracts during the search, after removing the duplicates. Handsearching references from retrieved articles did not add any additional study. Forty studies were excluded based on titles and abstracts, resulting in 20 full-text papers for detailed evaluation. Of those, 12 articles were excluded for the following reasons: (i) connectivity was not assessed with tractography (28, 29); (ii) DBS outcomes were not considered (30–33); (iii) no description of WMP was provided according to our defined criteria (34, 35); referred studies were duplicate in other selected articles (36). The eight studies included were published between 2016 and 2020 and originated from Spain (*N* = 1), Netherlands (*N* = 1), Germany (*N* = 3), United Kingdom (*N* = 1), and the United States (*N* = 2) (17, 20–23, 37–39) (Table 1).

**Table 1:** Characteristics of included studies.

**Reference**	**Country**	**Target**	**Number of patients**	**Follow-up (months)**	**Clinical Scale**	**Responders (≥35% improvement)**	**Mean improvement (%)**
Barcia et al. (38)	Spain	NAC	7	3	Y-BOCS	6	51.33 ± 20.98
Liebrand et al. (23)	Netherlands	ALIC	12	12	Y-BOCS	7	40.8 ± 26.87
Baldermann et al. (20)	Germany	ALIC/NAC	22	12	Y-BOCS	Not reported	31.0 ± 20.5%
Coenen et al. (21)	Germany	MFB	2	12	Y-BOCS	2	41.7 ± 11.79
Tyagi et al. (39)	United Kingdom	VC/VS/STN	6	12	Y-BOCS	6	73.83 ± 22.31
Hartmann et al. (37)	USA	ALIC/NAC	6	24	Y-BOCS	2	36.17 ± 31.82
Makris et al. (17)	USA	ALIC	1	6	Y-BOCS	1	35^*^
		Cologne – ALIC	Cologne – 22				Cologne 31.0 ± 20.5%
		Grenoble – STN	Grenoble – 14				Grenoble 41.2 ± 31.7%
Li et al. (22)	Germany	Madrid – NAC	Madrid – 8	3–12	Y-BOCS	Not reported	Madrid 47.8 ± 23
		London – ALIC + STN (4 DBS/pac)	London – 6 (total: 50)				London 50.0 ± 12.6%

*ALIC, anterior limb of the internal capsule; NAC, nucleus accumbens; MFB, medial forebrain bundle; VC/VS, ventral capsule/ventral striatum; STN, subthalamic nucleus; Y-BOCS, Yale-Brown Obsessive-Compulsive Scale; OCD, obsessive-compulsive disorder; DBS, deep brain stimulation*.

* *No mean improvement was provided because this was a single case report*.

### Target

DBS electrodes were implanted in the VC/VS (20, 37, 39), NAC (38), ALIC (17, 23), STN (39) and MFB (21). One study analyzed cohorts of patients implanted with electrodes in multiple targets, including the ALIC, STN, NAC, and ALIC plus STN (22). The active contacts in some NAC DBS trials were actually localized in the ventral portion of the ALIC.

### Participants

The number of patients per study ranged from 1 to 50. Five studies included <8 patients (17, 21, 37–39). One study included 12 patients (23). The highest number of patients in a single center was 22 (20). One of the reports analyzed four cohorts from different centers, including a total of 50 patients (22). Some of these patients might have also been reported in other individual articles: University Hospital Cologne (20), Hospital Clínico San Carlos Madrid (38) and University Hospital London (39) cohorts.

### Postoperative Follow-Up

Follow-up ranged from 3 to 24 months. Two studies assessed symptoms at 3–6 months (17, 38). Longer follow-up (12–24 months) was presented in six studies (20–23, 37, 39).

### Responders/Mean Improvement

In the studies pooled for analysis, 24 patients were considered to be treatment responders, defined in the literature as ≥35% improvement in postoperative Y-BOCS scores. Two articles only report the mean improvement rate in their cohorts (20, 22). Higher rates of improvement were reported by Tyagi et al. (39) (73.83 ± 22.31%) and Barcia et al. (38) (51.33 ± 20.98 %). A lower, but significant improvement was found in the trials published by Baldermann at al. (20) (31.0 ± 20.5%), Liebrand et al. (23) (40.8 ± 26.87%), Makris et al. (17) (35%), and Coenen et al. (21) (41.7 ± 11.79%). One study reported the mean improvement of each assessed cohort: Cologne 31.0 ± 20.5%; Grenoble 41.2 ± 31.7%; Madrid 47.8 ± 23%; London 50.0 ± 12.6%) (22) (Table 1).

### Imaging Acquisition

Most studies were performed in 3T MRI scans from Siemens Medical System (Erlangen, Germany) (17, 20, 21, 38, 39), or Philips Medical Systems (Best, The Netherlands) (23). One study used 1.5T MRI (37) (see Table 2).

**Table 2:** Characteristics of imaging acquisition, processing, and connectivity.

		**MRI Scanner**		**Diffusion acquisition**			**Diffusion post processing**		
**Reference**	**Number of patients**	**Strength of the main static magnetic field (T)**	**Manufacturer and version**	**Number of diffusion gradient directions**	**Diffusion b-value (s/mm^**2**^)**	**Voxel size (mm^**3**^)**	**Registration**	**Software**	**Fiber tracking method**	**Normatization to Structural data**
Barcia et al. (38)	7	3	Siemens Trio	64	500, 100, 0	2.3 isotropic	Preop T1WI, fMRI, Postop CT	Freesurfer, FSL (FLIRT, BET, FDT BedpostX, FDT Probtrackx (11 diffusion parameters)	Probabilistic (5,000 samples, max.steps: 2,000, min. step length: 0.5mm, curvature threshold 80°)	Not reported
Liebrand et al. (23)	12	3	Philips Ingenia	30/32	1,000, 0	1.8 × 1.8 × 3.0 (7/12) & 2.0 isotropic (5/12)	3T Preop T1WI, 1.5T with frame T1WI, Postop CT	FSL (probtrackx), ANTs	Probabilistic (5,000 samples; max. steps: 2,500; step length:0.5 mm, curve threshold 0.2)	Native MRI, MNI
Baldermann et al. (20)	10	3	Siemens Magnetom Prisma	90 (12 AP, 8 PA)	3,000	1.7 isotropic	3T Preop T1WI, Postop CT	DSI-Studio, Lead DBS	Deterministic (200,000 samples, 60°, step size: 0.86 mm, tracks length: 10 mm	MNI, HCP
Coenen et al. (21)	2	3	Siemens Trio Magnetom Prisma	61	1,000, 0	2.0 isotropic		StealthViz DTI (Medtronic)	Deterministic	Not reported
Tyagi et al. (39)	6	3	Siemens Magnetom	128 (R/L: ([128 + 7] × 2)	1,500, 0	1.5 isotropic	3T preop T1WI	FSL (Topup, Eddy, BET, FLIRT, FNIRT, BedpostX, ProbtrackX2)	Probabilistic (5,000 samples, curvature threshold = 0.2, step length = 0.5 mm)	MNI, HCN
Hartmann et al. (37)	6	1.5	Not reported	60	1,000	2.0 isotropic	1.5T preop T1WI	FSL (FLIRT, BET, FNIRT, BedpostX, ProbtrackX2), Freesurfer, MATLAB	Probabilistic (1,000 samples, step length 0.5 mm, curvature threshold ±80)	Oxford
Makris et al. (17)	1	3	Siemens Trio	60	700	1.93 isotropic	3T preop T1WI, postop CT	FSL (FNIRT)	Deterministic	MNI, HCP
Li et al. (22)	50 C: 22 G: 14 M: 8 L: 6	3	Preop MRI Not reported	Not reported	Not reported	Not reported	3T preop T1WI, 1.5T postop T1WI (11/14pac G + L), postop CT (remaining)	Lead-DBS (G, C, M), Medtronic (L), ANTSs, Lead-Connectome	Deterministic	MNI, HCP, HRAP

*MNI, Montreal Neurological Institute Standard structural space; HCP, Human Connectome Project at Massachusetts General Hospital, Normative connectome; ANT, Advanced Normalization Tools; HCN, The Human Central Nervous System: A Synopsis and Atlas; Oxford (Oxford Centre for Functional MRI of the Brain, Oxford, UK); HRAP, Petersen, M. V. et al. Holographic Reconstruction of Axonal Pathways in the Human Brain. Neuron 104, 1056-1064.e3 (2019); In Li et al., data from the following surgical centers was included: C, Cologne; G, Grenoble; M, Madrid; L, London*.

### Diffusion Imaging Acquisition Features

There was a wide variability range among diffusion acquisition parameters, as shown in Table 2. Regarding the number of diffusion gradient directions, half of the studies reported values between 60 and 64 directions (17, 21, 37, 38). Two studies used a higher number of directions: 90 and 128, respectively (20, 39). One study used a lower number: 32/30 (23). One article did not report this parameter (22).

The majority of studies used diffusion b-values of 1,000 s/mm^2^ or lower (17, 21, 23, 37, 38). Some reported b-values of 1,500 s/mm^2^ (39) and 3,000 s/mm^2^ (20), with a higher sensitivity for tract detection.

There was small variation in voxel size across studies, with high (2.3 mm^3^) (38), intermediate [2.0 mm^3^ (21, 37) and 1.93 mm^3^ (17)], and low isotropic values [1.7 mm^3^ (20), and 1.5 mm^3^ (39)] being reported. One study had a sample variation with seven out of 12 patients presenting a voxel size of 1.8 × 1.8 × 3.0 mm^3^, and 5 isotropic 2.0 mm^3^ (23). Barcia et al. (38) created volumes with no interslice gap. We could not retrieve additional information on slice gap, parallel image acquisition, or daily image quality assessments.

### Diffusion Connectivity Post Processing

Diffusion descriptive parameters, such as fractional anisotropy (FA), mean diffusivity (MD), and apparent diffusion coefficient (ADC) were not reported in the selected studies. One group mentioned the variability of these parameters, but only in controls (17). Additional details can be found in Table 2.

The preferred fiber tracking method was probabilistic reconstruction, usually performed with the FSL software (www.fsl.fmrib.ox.ac.uk) (23, 37–39). The deterministic method was conducted with a broader range of softwares, including the DSI-Studio (20), StealthViz DTI (Medtronic Navigation, Louisville, CO, USA) (21), and FSL (17).

Except for Liebrand et al. (23), who assessed the distances between active DBS contacts and WMP of interest (MFB or anterior thalamic radiation) in native DTI-MRI space, other studies analyzed their data in normative connectomes, as described below.

The method of activation volume tractography (AVT) was used in four studies (20, 22, 38, 39). Briefly, a volume of activated tissue around the stimulating contact is modeled using a four-compartment mesh algorithm that includes gray matter, white matter, electrode parameters, and insulated parts. This algorithm is embedded in the Lead-DBS software (40) or in the Medtronic SureTune software™. Deterministic or probabilistic methods are then applied using the center of this volume as seeds, subsequently building fibers running within this volume.

### WMP and Cortical Areas Across the Studies

Hartmann et al. (37) evaluated six patients and classified them in three groups: best responders (at least 50% Y-BOCS reduction); no-responders (Y-BOCS reduction lower than 10%); or moderate responders (Y-BOCS reduction between 10 and 50%). They showed an association between best clinical outcome and activation of the anterior part of the right middle frontal gyrus (MFG), a region comprising Brodmann areas 9 and 46. Although, similar activation was found within this same region in non-responders, this group showed a larger number of fibers projecting to the right thalamus, NAC and the orbital segment of the right inferior frontal gyrus (Brodmann area 47), which corresponded to the anterior ventrolateral PFC and lateral OFC.

The inter-subject variability of fibers within the ALIC was addressed in one study showing that the medial (mOFC) and the lateral orbitofrontothalamic connections (lOFC) varied significantly across 29 healthy subjects. Though running in parallel, these tracts resembled a “cloud” intertwining within the ALIC (37). The authors also evaluated the position of DBS contacts in one patient who improved 35% after VC/VS DBS and found that neighbor DBS contacts, although very close to each other, could engage distinct fiber tracts (mOFC or lOFC).

Coenen et al. (21) directly stimulated the supero-lateral branch of the MFB in OCD. Using deterministic tractography, they targeted this tract bilaterally in two patients who improved by 35 and 50% at 12 months after surgery. The involvement of the MFB in the effects of DBS was strengthened by another study that assessed 12 patients receiving bilateral DBS in the ventral ALIC (23). Using probabilistic tractography on patient-specific space, the authors showed that better clinical responses were found in individuals whose active DBS contacts were closer to the MFB than to the anterior thalamic radiation (ATR). They further assessed the anatomical location of the active contacts within a standard anatomical space (Montreal Neurological Institute – MNI space) and showed a similar location in responders and non-responders.

Baldermann et al. (20) evaluated the structural connectivity of ALIC/NAC DBS in 22 patients using AVT. The authors found that WMPs associated with positive outcomes crossed the ALIC dorsal to the NAC and connected the medial PFC with the thalamus. These streamlines within the fronto-thalamic radiation could be discriminated with the use of electrodes leading to optimal vs. suboptimal responses. In contrast, WMPs associated with negative outcomes were suggested to run within the MFB, the posterior limb of the anterior commissure (AC) and the inferior lateral fascicle (ILF). In line with Hartmann's findings (37), the authors have shown a significant correlation between symptom reduction and connectivity of the volume of tissue activated (VTA) around the stimulating contacts and the right MFG.

A recent study used AVT and deterministic methods to analyze therapeutic DBS networks in 50 patients implanted in different brain targets at four DBS centers (22). The authors carried out structural connectivity between VTAs and cortical regions using the HCP template and weighted the streamlines according to their ability of discriminating between good and poor responders. These analyses returned a WMP running in the dorsal ALIC, that connected the dorsal cingulate cortex to the STN and the mediodorsal nucleus of the thalamus (MD-Th). These fibers associated positively with clinical outcome and showed similar spatial distribution, irrespective of the stimulated target, thus proposing a single tract target for DBS effects on OCD. The existence of multiple therapeutic pathways, however, was proposed by a randomized clinical trial, which showed that VC/VS DBS had a greater effect on depression, whereas anteromedial STN DBS was associated with more striking improvements on cognitive flexibility (39). Accordingly, probabilistic AVT tractography showed that VATs around the STN were connected to the lateral OFC, dorsal anterior cingulate, dorsolateral PFC and MFB, whereas, VATs around VC/VS were connected to the medial OFC, MD-Th, amygdala, hypothalamus and habenula. Finally, Barcia et al. (38) used probabilistic AVT in seven patients implanted in the NAC to assess the structural connectivity between VTAs around all DBS contacts and PFC regions activated in a functional MRI paradigm (fMRI), during which provocative images were shown. The authors concluded that the VTAs yielding the best clinical responses not only had different anatomical positions in individual patients, but also showed higher connectivity with PFC areas activated during the paradigm. We emphasize that, although most of the patients studied by Li et al. were also reported in other studies (i.e., Balderman et al., Barcia et al., and Tyagi et al.), the connectomic analyses (the focus of this review) differed significantly across the authors. This prompted us to analyze each study individually.

**Figure 2:**
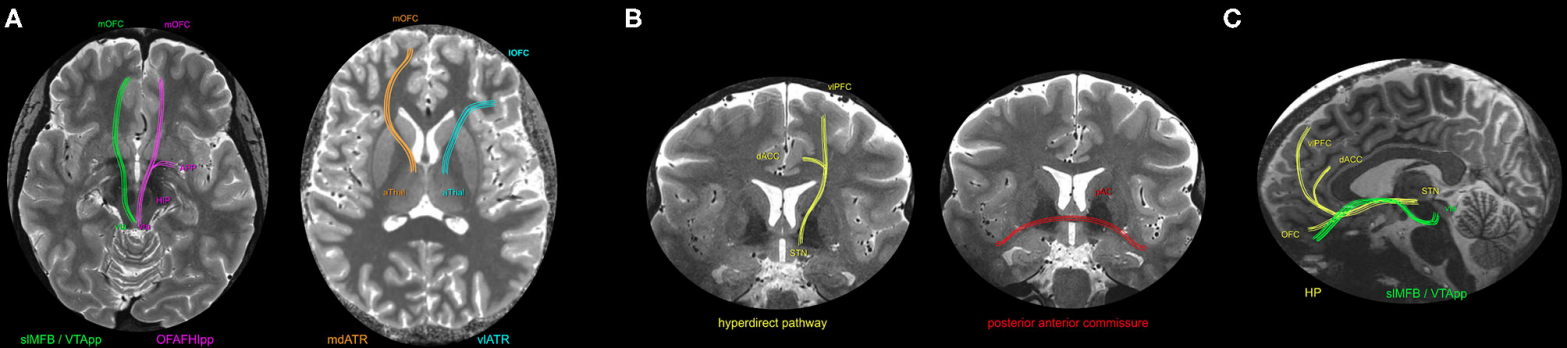
Schematic representation of the WMP possibly modulated by DBS in OCD patients, overlaid in a 7T MP2RAGE T1 map (Siemens, Magnetom, Germany, from PISA - FMUSP, São Paulo). **(A)** Axial planes representing four WMP: In green, the superolateral middle forebrain bundle (slMFB) - named afterwards as ventral tegmental area projection pathway (VTApp) - projects from the ventral tegmental area (vta) to mOFC (medial orbitofrontal cortex) in a ventral level (21, 23). In pink, the orbitofrontal amygdalofugal habenulo-interpeduncular pathway (OFAFHIpp), from the vta to amygdala and mOFC, was mentioned in (39). In orange, the medial dorsal anterior thalamic radiation (mdATR), from anterior thalamus to mOFC, was cited in (17, 20, 23, 37). In blue, the ventrolateral anterior thalamic radiation (vlATR), mentioned in (17, 21, 37). **(B)** Coronal plane, representing two WMP: In yellow, the hyperdirect pathway (HP) comprises fibers from the subthalamic nucleus (STN) to both dorsal anterior Cingulate cortex (dACC) and ventrolateral prefrontal cortex (vlPFC) (22, 38, 39). In red, the posterior limb of the anterior commissure, connecting bilateral temporal cortices (22). **(C)** Sagittal plane, representing two of the above mentioned WMP: slMFB/VTApp and the HP, to optimize the 3D comprehension.

Figure 2 shows a scheme of the main fibers described above.

## Discussion

First proposed in 1999 (12), DBS has been shown as a safe and efficient therapy for medically-resistant OCD patients. Despite the substantial benefits, it is still unclear why 30–50% of patients show no meaningful response after surgery, thus justifying additional studies focused on target refinement. In this scenario, tractography and connectivity analyses have been proposed for the investigation of the therapeutic networks possibly involved in DBS effects (41). In OCD, a few studies have argued that the MFB (21, 23), the fronto-thalamic pathway (20, 22) and the hyperdirect PFC-STN tract (22) constitute potential WMPs involved in therapeutic effects of DBS. Furthermore, a unified connectomic hypothesis was proposed (22) despite data suggesting that stimulation delivered to different brain regions may be associated with distinct outcomes (39) and engage different tracts and cortical areas (38, 39). Finally, significant inter-individual differences have been shown in regard to the anatomy of fibers connecting the PFC and the thalamus, thereby emphasizing the importance of patient-specific images during DBS for OCD (17).

Dysfunctions in different neurocircuitries modulated by DBS have been proposed to play a role in the pathophysiology of OCD endophenotypes (42). For example, changes in the functional connectivity between the amygdala and the ventromedial PFC were found after ALIC DBS, which may explain some effects on fear and anxiety (43). Moreover, the inferior frontal gyrus (IFG) and the STN mediate response inhibition, a function that is often impaired in OCD. Increased power in theta activity - a potential marker of response inhibition - was demonstrated after STN DBS and was correlated with symptom reduction (44). Increased theta oscillations in prefrontal regions, including the IFG, were also observed after VC/VS DBS during an inhibition task (45). Finally, the modulation of reward circuitries involving the NAC and the MFB may improve fronto-striatal dysfunctions linked to the pathophysiology of OCD (21). Despite not being the focus of this review, DTI-MRI was used to investigate WMP abnormalities in OCD patients (24, 46). In this aspect, most of the studies have shown reduced WMP connecting the anterior cingulate with the ventral BG, consistent with previous models from functional MRI. Further, structural connectivity changes were also reported in other brain networks, which vary as a function of both clinical characteristics and pharmacologic/psychotherapeutic interventions (46).

Since its proposal, DBS was performed on several targets with a good level of efficacy (47). When compared to DBS in Parkinson's disease, where 92.5% of the patients were satisfied, 95% would recommend, and 75% felt it provided symptom control after 10 years (48), DBS for OCD is associated with a relatively poor response in 40–50% of patients (49, 50). Factors that may explain this lower rate of efficacy include the lack of clinical variables and predictive biomarkers, as well as a failure to engage crucial therapeutic networks for different symptoms. Concerning this latter issue, the use of patient-specific connectomic analyses may improve the results obtained with the current surgical techniques based on fixed landmarks.

The results reported in this systematic review lend further support to the notion that tractography may be a complimentary method for surgical targeting in OCD, since (i) fibers that connect the PFC to the ventral striatum and thalamus nuclei seem to be involved in dysfunctions of reward and decision-making processes in OCD (7, 17, 44, 45); (ii) electrical stimulation of specific WMP may yield good or poor clinical responses (21, 23, 37, 38); (iii) responders and non-responders to DBS may be predicted by the activation of particular WMPs (22, 37); (iv) connectivity between fibers around DBS contacts and some PFC regions (e.g., the anterior portion of the right MFG) may be associated with good clinical response (22, 23); and (v) surgical outcome may improve with an individualized network approach taking into account specific clinical patterns (38, 39).

Despite the potential use of tractography in DBS for OCD, no consensus has been reached about which WMP should be modulated. The superolateral branch of the MFB was first targeted for refractory depression (51) and later proposed in OCD (21). Coenen et al. (21) have shown a significant improvement in two patients receiving DBS directly in the MFB. Electrical stimulation of this WMP may change the functional connectivity between the amygdala and the ventromedial PFC, which may explain some of the DBS effects on fear and anxiety (42, 43). Subsequently, Liebrand et al. (23) studied 12 patients receiving bilateral DBS in the ventral ALIC and reported greater clinical improvement when stimulating contacts closer to the MFB than to the ATR. This was the only study including more than 10 patients that assessed DTI-MRI in native space, thus taking into account the interindividual anatomical variance of WMP. This study established that the anatomical position of DBS contacts in the MNI space did not differ between responders and non-responders, a conclusion which was later toned down by the authors, since anatomical distinctions across subjects was not consider.

Different results were shown by Balderman et al. (20), who evaluated WMP running within the VTAs of 22 patients implanted with DBS in the ALIC/NAC. Accordingly, fronto-thalamic streamlines were associated with better outcomes, while poor results were found when VTAs encompassed the MFB. Similar conclusions were found with the use of two different normative connectomes in cohorts of 10 OCD patients and 32 healthy subjects. The relevance of PFC-thalamic projections was further highlighted in the study by Li et al. (22) who assessed data from 50 patients in four European centers. The authors concluded that fibers associated with better outcomes in OCD were those projecting from the dorsal cingulate cortex to the STN (the limbic hyperdirect pathway) and the MD-Th nucleus. They also discussed some discrepancies regarding the anatomical position of the MFB according to previous anatomical and more recent DTI-MRI studies. Based on this aspect, Coenen et al. (31) have recently changed the term MFB to ventral tegmental area projection pathway (vtaPP), which run dorsally to the classical MFB.

Similar to other diseases where different symptoms are related to different connectivity patterns (52), discrete types of OCD symptoms (e.g., checking and contamination) seem to be associated with the activation of different PFC regions (53). Along this line, Barcia et al. (38) showed that the most effective DBS contacts had stronger connectivity with specific PFC areas activated after the presentation of images that evoked similar clinical symptoms. Concurrent results came from Tyagi et al. (39) who showed distinct networks recruited following VC/VS and STN DBS, the former impacting predominantly depressive and the latter cognitive symptoms. A complementary hypothesis was recently proposed by Li et al. (22), who stated that (i) the same WMP (the limbic hyperdirect tract) would work as a therapeutic network for common symptoms across different subjects, irrespective of the stimulated target; (ii) other WMPs not shared across patients and targets would be associated with a reduction in symptoms of specific OCD endophenotypes. Therefore, future studies are expected to focus on symptoms rather than diseases, or cluster patients in multiple homogeneous groups in order to shed light on therapeutic networks.

Most of the patients included in the revised reports have used normative connectomes from healthy subjects to study fiber tracts, except for Coenen et al. (21), Liebrand et al. (23), and Barcia et al. (38). This approach has clear limitations, since it neither accounts for anatomical variations across subjects nor considers changes potentially introduced by diseases (54). According to tractography studies, distinct WMPs coursing through the ALIC are functionally segregated, connecting different parts of the PFC with the thalamus, the ventral tegmental area and the STN (18, 31). Moreover, anatomical aspects of these fibers have been shown to vary across subjects (17, 18). This may explain why results of ALIC DBS differ from patient to patient and highlight the need of patient-specific neuroimaging strategies for surgical targeting (54, 55). Despite these limitations, normative connectomes provide high-quality images and enable mechanistic assumptions, not to mention structural connectivity hypothesis in patients lacking high-quality DTI-MRI (40).

Important limitations of our report include the small number of studies and patients. Although, neurosurgery for mental illness has been conducted for several decades (56), the number of OCD patients treated with DBS is relatively small, especially when compared to movement disorders (57). Thus, any hypothesis on therapeutic OCD networks has to be considered with caution and would need to be substantiated by future multicentric studies with a larger sample size. Another important limitation is related to methodological aspects of DTI, including the variability in data acquisition and differences in the algorithms used for WMP reconstruction (27). We compared studies that had differences in MRI manufacturers, phasing encoding distortions, and corrections mechanisms. This resulted in different images, even when obtained with identical parameters (58, 59). The inclusion of different magnetic fields (1.5T and 3T) not only introduces effects on signal to noise ratio, but also on distortions due to eddy currents, magnetic susceptibility and chemical shift artifacts, thereby, affecting the quality of images (59, 60). In these studies, estimating the fiber orientation was based on the dominant vector (tensor), the DTI. More accurate technical options could reduce some of these limitations (61). Some of such techniques include current advanced acquisitions based on the orientation distribution function (ODF), which exhibits similar accuracy as the Q-ball imaging (QBI) in High Angular Resolution Diffusion Imaging (HARDI) (59, 60, 62). HARDI has a higher number of gradient directions than DTI and may have b-values over 1,000, diffusion spectrum imaging (DSI) which uses multiple b-values of up to 7,000 s/mm^2^ or higher to sample q-space in a grid fashion, and multi-shell acquisitions that acquire multiple b-values (sampling spherical shells in q-space) (62). Most recent studies consider High Definition Fiber tracking (HDFT) as a good choice of DTI-MRI resolution, based on the fact that data is acquired from DSI and processed by generalized q-sampling imaging (GQI) (63). The acquisition may also be improved with phase encoding gradients in anterior-posterior and posterior-anterior directions. Although, it doubles the acquisition time, it reduces the spatial distortions in the echo-planar imaging (EPI) diffusion MRI scan which can affect WMP in the order of 2 mm, mainly in regions near air-bone interfaces (59, 60). The different ways of analyses may also impact the results, as deterministic or probabilistic estimation, with whole-brain tract-based spatial statistics (TBSS) and volume of interest (VOI) (60).

Despite the above-mentioned limitations, this systematic review shows that tractography may contribute to surgical targeting and allow the assessment of potential networks involved in OCD. Future studies with high-quality acquisition may increase the DTI-MRI accuracy, and migrate from normative connectomes to individualized data. Furthermore, for ideal WMP localization, comparison to postmortem studies with iron tracers and polarization-sensitive optical coherence tomography, could improve DTI-MRI technical choices for next studies.

## Conclusion

The studies in this systematic review support the hypothesis that a connectomic approach may assist conventional methods of DBS targeting and further reduce OCD symptoms in patients undergoing the procedure. The stimulation of specific networks may allow an optimal delivery of current tailored to the patients' clinical needs. The association of functional to structural methods of connectivity, allied to recent improvements in hardware technology, such as closed-loop and directional leads, shall embody high-definition medicine in the field of Neurosurgery for OCD and other disorders. We caution, however, that despite the promising results of initial tractography/connectivity studies, the use of these approaches in OCD patients treated with DBS is at an early stage. Overall, no conclusive remarks could be drawn from the handful of papers pooled in our review. Additional studies with a larger sample size and the prospective use of tractography/connectomic strategies for targeting are still necessary for a better appraisal of the role of these techniques in the field.

## Data Availability Statement

The original contributions presented in the study are included in the article/supplementary material, further inquiries can be directed to the corresponding author/s.

## Author Contributions

All authors listed have made a substantial, direct and intellectual contribution to the work, and approved it for publication.

## Conflict of Interest

The authors declare that the research was conducted in the absence of any commercial or financial relationships that could be construed as a potential conflict of interest.

## Abbreviations

ALIC: anterior limb of internal capsule
ATR: anterior thalamic radiation
AVT: activation volume tractography
DBS: deep brain stimulation
DTI: diffusion tensor imaging
DTI-MRI: magnetic resonance imaging diffusion analyses
fMRI: Functional Magnetic Resonance Imaging
MD-Th: mediodorsal nucleus of the thalamus
MFB: medial forebrain bundle
MFG: middle frontal gyrus
MRI: Magnetic Resonance Imaging
NAC: Nucleus Accumbens
OCD: obsessive-compulsive disorder
OFC: orbitofrontal cortex
PFC: prefrontal cortex
STN: subthalamic nucleus
VTA: volume of tissue activated
vtaPP: ventral tegmental area projection pathway
VC/VS: ventral capsule &#8211
ventral striatum
Y-BOCS: Yale-Brown Obsessive-Compulsive Scale
WMP: white matter pathways.

## Appendix 1. Pubmed Search Strategy

#1. MeSH descriptor: [Obsessive-compulsive disorder] explode all trees.

#2. MeSH descriptor: [Deep Brain Stimulation] explode all trees.

#3. MeSH descriptor: [Diffusion Tensor Imaging] explode all trees.

#4. [(tractography or diffusion tractography or diffusion analys*)].

#5 MeSH descriptor: [White Matter] explode all trees.

#6. MeSH descriptor: [Connectome] explode all trees.

#7. #1 and #2

#8. #3 or 4 or #5 or #6.

#9. #7 and #8.
